# Mutation of the *PIK3CA* oncogene in human cancers

**DOI:** 10.1038/sj.bjc.6602970

**Published:** 2006-01-31

**Authors:** B Karakas, K E Bachman, B H Park

**Affiliations:** 1 The Sidney Kimmel Comprehensive Cancer Center at Johns Hopkins, The Johns Hopkins University School of Medicine, Department of Oncology, Baltimore, MD 21231, USA; 2 The Greenebaum Cancer Center, University of Maryland School of Medicine, Baltimore, MD 21201, USA

**Keywords:** PI3K, PIK3CA, p110*α*, somatic mutations

## Abstract

It is now well established that cancer is a genetic disease and that somatic mutations of oncogenes and tumour suppressor genes are the initiators of the carcinogenic process. The phosphatidylinositol 3-kinase signalling pathway has previously been implicated in tumorigenesis, and evidence over the past year suggests a pivotal role for the phosphatidylinositol 3-kinase catalytic subunit, PIK3CA, in human cancers. In this review, we analyse recent reports describing *PIK3CA* mutations in a variety of human malignancies, and discuss their possible implications for diagnosis and therapy.

Recently, somatic mutations in many different human cancers were discovered in the gene encoding for the phosphatidylinositol 3-kinase (PI3K) catalytic subunit, *PIK3CA*. In this review, we will be analysing and consolidating these findings, and discuss their possible implications in cancer progression and therapy. Due to the focused nature of this review, the PI3K pathway and its biochemical signalling properties will not be discussed in detail. The reader is directed toward several excellent recent reviews for a more comprehensive analysis of this pathway (Hennessy *et al*, 2005; Wymann and Marone, 2005).

## PHOSPHATIDYLINOSITOL 3-KINASE OVERVIEW

The PI3Ks are heterodimeric lipid kinases composed of catalytic and adaptor/regulatory subunit variants encoded by separate genes and alternative splicing. Phosphatidylinositol 3-kinases are important regulators of cellular growth, transformation, adhesion, apoptosis, survival and motility (Volinia *et al*, 1994; Fruman *et al*, 1998; Cantley, 2002). The PI3K family of enzymes are organised under three main classes (class I, II and III) and various subgroups have been categorised based on their primary structure, substrate specificity and regulation (Vivanco and Sawyers, 2002).

**Figure 1:**
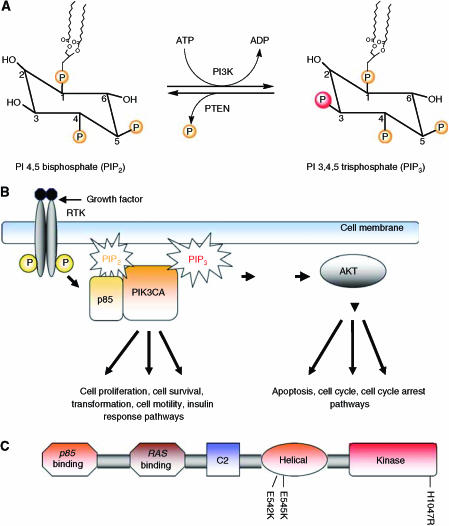
(**A**) The main reaction catalysed by PI3K: phosphatidylinositol (PI) 4,5 bisphosphate (PIP_2_) to phosphatidylinositol (PI) 3,4,5-triphosphate (PIP_3_). (**B**) PI3K is activated upon ligand binding to a receptor tyrosine kinase (RTK), which then activates the regulatory subunit (p85) to bind the catalytic p110*α* subunit. This ultimately triggers various downstream signalling cascades resulting in cell survival, apoptosis, transformation, metastasis, and cell migration. (**C**) Schematic representation of PIK3CA (p110*α* catalytic subunit of PI3K) and its functional domains with the most common somatic mutations, E542K, E545K and H1047R within the helical and kinase domains indicated.

Both the catalytic and regulatory subunits of the human *PI3K* gene were cloned by Volinia *et al* (1994) and the overall sequence was found to be highly homologous to the bovine and yeast *PI3K* genes. The activation of PI3K results in the generation of the second messenger, phosphatidylinositol 3,4,5 trisphosphate (PIP_3_) from phosphatidylinositol 4,5 bisphosphate (PIP_2_) (Figure 1A). The activation of PI3K by a growth factor bound (activated) receptor tyrosine kinase (RTK) and subsequent production of PIP_3_ drives the various downstream pathways that regulate a number of cellular functions including those involved in tumour development and progression (Figure 1B).

## PHOSPHATIDYLINOSITOL 3-KINASE AND HUMAN CANCER

The kinase activity of PI3K was first reported to be associated with viral oncoproteins (Cantley *et al*, 1991). Subsequent studies employing mouse knockouts of both the regulatory and catalytic subunits of PIK3 resulted in a number of deficits including embryonic lethality, B cell defects, liver necrosis and colorectal cancer (Katso *et al*, 2001). Other investigations showed that the amplification of the *PI3K* locus as well as deletions of short nucleotide sequences resulted in elevated lipid kinase activity of the p110*α* catalytic subunit of PI3K (PIK3CA) in various cancer types with the implication that *PI3K* was functioning as an oncogene (Volinia *et al*, 1994; Shayesteh *et al*, 1999; Ma *et al*, 2000; Katso *et al*, 2001; Migozuchi *et al*, 2004; Pedrero *et al*, 2005). The PIK3CA p110*α* catalytic subunit of PI3K will be highlighted in this review due to the recent alterations of this protein found in primary human cancers. *PIK3CA* is a 34 kb gene located on chromosome 3q26.3 that consists of 20 exons coding for 1068 amino acids yielding a 124 kDa size protein (Figure 1C). Gene amplifications, deletions and more recently, somatic missense mutations in the *PIK3CA* gene have been reported in many human cancer types including cancers of the colon, breast, brain, liver, stomach and lung. These somatic missense mutations were proposed to increase the kinase activity of PIK3CA contributing to cellular transformation. The first of these mutational reports was published by Samuels *et al* (2004). In this seminal paper, the authors initially analysed the sequence of eight *PI3K* and eight *PI3K*-like genes in a relatively small number of primary colorectal tumours and discovered that *PIK3CA* was the only gene harbouring somatic mutations. They subsequently expanded their sample size, which included tissues from primary tumours of the colon, brain, breast, stomach and lung. Their results verified their initial observations and demonstrated that somatic mutations were found in all of these tissues at varying frequencies. Notably, colorectal, brain and gastric cancers were found to have a high rate of *PIK3CA* gene mutation with frequencies of 32, 27 and 25%, respectively. Breast and lung cancers had a relatively low rate of *PIK3CA* mutations (8 and 4% respectively), although the sample size of all cancer types was relatively small (*n*=12–24) with the exception of colorectal cancers (*n*=234). These somatic missense mutations were scattered across most of the exons, but were predominantly found in the kinase and helical domains of the PIK3CA subunit (Table 1). Of note, ‘hotspot’ or frequently recurring mutations were found in exon 9 (G1624A:E542K) and exon 20 (A3140G:H1047R) in this analysis. Based on all sequencing data (Table 1), there now appear to be three hotspots mutations within *PIK3CA*: H1047R, E542K and E545K. Bachman *et al* (2004) expanded this report using a larger sample set consisting of primary breast cancers and breast cancer cell lines. Their data demonstrated that on average 25% of breast cancers harbour missense mutations in either the kinase, helical or p85 binding domains, although it should be noted that only the three exons corresponding to these domains were sequenced in their analysis. Many other studies followed, examining *PIK3CA* mutations in various cancer types (Table 1). Campbell *et al* (2004) sequenced all of the 20 coding exons of *PIK3CA* from primary tumour samples of breast, ovarian and colorectal cancers and reported new mutations found in exons 6, 7 and 9, as well as mutations previously reported by others. They reported a *PIK3CA* mutation frequency of 18.8% in colorectal cancers and among 70 breast cancer samples, they noted a mutation frequency of 40%, which is thus far the highest reported in any cancer type (Table 1). The frequency of ovarian cancers was reported as 6%, but of note, mutations clustered according to the histologic subtype with endometrioid and clear cell variants having a much higher rate than serous and mucinous ovarian cancers. In both the studies by Bachman *et al* and Campbell *et al*, no association was noted between the presence of *PIK3CA* mutations with other prognostic/clinical features of breast cancer, including histologic subtype, oestrogen/progesterone receptor expression, Her2/neu receptor status, axillary lymph node positivity, grade and/or stage of the tumour. This is in contrast to a more recent analysis by Saal *et al* (2005) where these authors examined a total of 292 primary breast cancers and found an overall mutation rate of 26%. In this study, the authors described a statistically significant correlation between the presence of *PIK3CA* mutations and the presence of nodal metastases, oestrogen/progesterone receptor positivity and Her2/neu receptor overexpression/amplification. They also demonstrated a statistically significant correlation between the presence of *PIK3CA* mutations and the presence of PTEN expression, an intriguing finding given the known roles of these two pathways and similar findings in brain cancers (see below). As described by Saal *et al*, variations in sample size likely account for the discrepancies between their study and those of Bachman *et al* and Campbell *et al*, although regional bias in the tumour samples is still a possibility given that roughly half their samples were from a Swedish cohort that included almost exclusively Stage II breast cancers. Additionally, Levine *et al* (2005) sequenced *PIK3CA* exons 9 and 20 in 198 ovarian and 72 breast cancers using primary tissue samples and found an overall mutation rate of 12% for ovarian cancers and 18% for breast cancers, although no correlation with histologic subtypes and/or clinical/prognostic indicators were found for either type of cancer. Finally, Broderick *et al* (2004) sequenced the *PIK3CA* gene in 285 brain tumours and found a mutational rate of 5%, which was significantly lower than the rate originally reported by Samuels *et al* (2005). While the majority of their mutations were in known hotspot regions of the gene, these authors also found that *PIK3CA* mutations were restricted to certain histologic subtypes. They also showed in a limited analysis that *PIK3CA* mutations were mutually exclusive with mutations of the tumour suppressor *PTEN*, suggesting that tumorigenic signalling through this pathway can occur either through activation of PIK3CA *or* inactivation of PTEN. Given the ubiquitous nature of *PIK3CA* mutations in human cancers and the conflicting results of the above studies, the association of *PIK3CA* mutations with other clinical and histologic parameters is still not definitively known.

Another recent study (Lee *et al*, 2004) demonstrated a very high rate (36%) of *PIK3CA* somatic mutations in liver cancer. In the same study, these authors also analysed tissues from breast, gastric and lung cancers using a relatively high sample size and found mutation rates similar to other studies (Table 1). Interestingly, the authors also found one *PIK3CA* mutation out of 88 acute leukaemias (mutation rate=1.1%) that were analysed in this study, suggesting that *PIK3CA* mutations are not limited to solid tumours of epithelial origin. An analysis of *PIK3CA* somatic mutations and amplifications in thyroid cancers (Wu *et al*, 2005b) did not reveal any *PIK3CA* mutations; however, this group did find *PIK3CA* gene amplification in 12% of thyroid adenomas, 5% of papillary thyroid cancers, 24% of follicular thyroid cancers and 71% of thyroid cancer cell lines. More recently, there has been a report of somatic mutations in genes (i.e. PDK1, AKT2 and PAK4) downstream of the PI3K signalling pathway (Parsons *et al*, 2005).

Although the frequency of mutations and the discovery of hotspot heterozygous mutations strongly argue for the importance of PIK3CA in the carcinogenic process, functional analysis of these mutations has also been performed to confirm this supposition. Overexpression of common hotspot *PIK3CA* mutations, as well as gene deletion experiments using somatic cell knockouts, has demonstrated that these mutations are in fact oncogenic (Ikenoue *et al*, 2005; Kang *et al*, 2005; Samuels *et al*, 2005). Kang *et al* (2005) overexpressed cDNAs containing the common *PIK3CA* mutations, E542K, E545K, and H1047R, in chicken embryo fibroblasts. Their study demonstrated that overexpression of these mutant PIK3CA proteins led to cellular transformation with concomitant phosphorylation of proteins in the AKT pathway. Through the use of somatic cell knockouts, Samuels *et al* (2005) reported that mutation of the *PIK3CA* kinase domain in the HCT116 colon cancer cell line, and mutation of the helical domain in the DLD1 colon cancer cell line, resulted in increased activity of the PIK3CA enzyme as manifested by increased cell signalling, cell growth and invasion. Another functional study examining the E542K, E545K and H1047R hotspots was reported by Ikenoue *et al* (2005). These authors found that an increase in PIK3CA kinase activity and cellular transformation occurred when the above-mentioned mutant *PIK3CA* sequences were introduced into mouse NIH 3T3 cells.

By combining the copious amount of sequencing data over the past year, we find that the *PIK3CA* gene is mutated on average in 15% of human cancers, although there is obviously great variability in the tissue type, that is, colon *vs* breast *vs* lung (Table 1). In most tissue types, mutations predominantly cluster within the three aforementioned hotspots: E542K, E545K and H1047R (Figure 1C). It is now evident that cancers of the liver, colon and breast harbour the most *PIK3CA* mutations with average mutational frequencies (across the reported studies) of 36, 26 and 25%, respectively (Table 1). The mutational studies that are summarised in Table 1 do reveal some conflicting results, however, and as previously mentioned these are likely due to a number of factors including geographical variation/influence, sample source preservation and methods used for DNA isolation. However, despite these discrepancies, the high frequency of *PIK3CA* mutation and the discovery of hotspot mutations have important clinical implications for diagnosis, prognosis and therapy. For example, using PCR and sequencing of hotspot mutations, increased diagnostic sensitivity of cancer may be possible in situations of histologic ambiguity. As a case in point, detection of disease positive nodes in breast cancer may benefit from this type of molecular diagnostic test. The detection and prognostic significance of micrometastatic nodal disease in breast cancer has yielded conflicting and controversial results, and so far, no definitive data have been presented (Sakorafas *et al*, 2004; Colleoni *et al*, 2005; Kuijt *et al*, 2005). This may be due in part to the lack of specificity used in these studies to detect cancerous cells within normal appearing lymph nodes. One could envision that if a woman's primary breast cancer harboured a *PIK3CA* mutation, then that same mutation could be screened for in her axillary lymph nodes that were otherwise histologically normal, using recently developed technologies that allow for the detection of minute amounts of mutant DNA molecules (Dressman *et al*, 2003; Diehl *et al*, 2005). From a prognostic standpoint, long-term prospective, blinded randomised trials could be performed to determine if the presence or absence of *PIK3CA* mutations have any correlation with clinical outcome in various cancer types. This would then allow for the clinician to predict with a fair amount of certainty whether or not cancers harbouring these mutations would be more or less aggressive and could therefore influence decisions regarding additional systemic therapies. Finally, targeted therapies such as Imatinib mesylate (anti-BCR/ABL and cKIT), Gefitinib and Erlotinib (anti-EGFR) that appear to impart a high degree of specificity for translocated/mutated oncogenes give hope that therapies targeted specifically against mutant PIK3CA can be developed (Druker *et al*, 2001; Lynch *et al*, 2004; Paez *et al*, 2004). Given the high degree of *PIK3CA* mutations in human cancers, this could have a tremendous impact on eliminating the morbidity and mortality of malignant diseases.

## Figures and Tables

**Table 1:** Somatic mutations of PIK3CA in cancer types reported since 10/2005

**Cancer**	**% PIK3CA mutation^a^**	**Sample source (primary tissue *vs* cancer cell line)**	**Exon mutated**	**Functional domain**	**Reference**
Liver	35.6 (26/73)	Primary	9 and 20	Helical and kinase	Lee *et al* (2004)
					
Total liver 36% (26/73)					
					
Breast	33.3 (4/12)	Cell lines	9 and 20	Helical and kinase	Bachman *et al* (2004)
Breast	21.4 (9/42)	Primary	1, 9 and 20	p85, helical and kinase	Bachman *et al* (2004)
Breast	18.1 (13/72)	Primary	9 and 20	Helical and kinase	Levine *et al* (2005)
Breast	40.0 (28/70)	Primary	6, 7, 9 and 20	C2, helical and kinase	Campbell *et al* (2004)
Breast	20.7 (19/92)	Primary	9 and 20	Helical and kinase	Wu *et al* (2005a)
Breast	8.3 (1/12)	Primary	20	kinase	Samuels *et al* (2004)
Breast	33.3 (5/15)	Cell lines	9 and 20	Helical and kinase	Wu *et al* (2005a)
Breast	26.9 (25/93)	Primary	9 and 20	Helical and kinase	Lee *et al* (2004)
Breast	28.0 (14/50)	Cell lines	1, 9 and 20	p85, helical and kinase	Saal *et al* (2005)
Breast	26.4 (77/292)	Primary	1, 4, 7, 9, 13, 18, 20	p85, C2, helical and kinase	Saal *et al* (2005)
					
Total breast 26% (195/750)					
					
Colon	31.6 (74/234)	Primary	1, 2, 4, 7, 9, 18 and 20	P85, C2, helical and Kinase	Samuels *et al* (2004)
Colon	13.6 (14/103)	Primary	9 and 20	Helical and kinase	Velho *et al* (2005)
Colon	18.8 (6/32)	Primary	9 and 20	Helical and kinase	Campbell *et al* (2004)
					
Total colon 25% (94/369)					
					
Ovarian	12.1 (24/198)	Primary	9 and 20	Helical and kinase	Levine *et al* (2005)
Ovarian	6.0 (11/182)	Primary	9 and 20	Helical and kinase	Campbell *et al* (2004)
					
Total ovarian 9% (35/380)					
					
Gastric	25.0 (3/12)	Primary	18 and 20	Kinase	Samuels *et al* (2004)
Gastric	10.6 (5/47)	Primary	9 and 20	Helical and kinase	Velho *et al* (2005)
Gastric	6.5 (12/185)	Primary	9 and 20	Helical and kinase	Lee *et al* (2004)
Gastric	4.3 (4/94)	Primary	9 and 20	Helical and kinase	Li *et al* (2005)
					
Total gastric 7% (24/338)					
					
Brain	26.7 (4/15)	Primary	4, 5 and 13	C2 and helical	Samuels *et al* (2004)
Brain	4.6 (13/285)	Primary	9 and 20	Helical and kinase	Broderick *et al* (2004)
					
Total brain 6% (17/300)					
					
Lung	1.3 (3/229)	Primary	9 and 20	Helical and kinase	Lee *et al* (2004)
Lung	4.2 (1/24)	Primary	9	Helical	Samuels *et al* (2004)
					
Total lung 2% (4/253)					
					
Leukaemia	1.1 (1/88)	Primary	9	Helical	Lee *et al* (2004)
					
Total leukaemia 1% (1/88)					
Total cancers reported 15% (382/2551)					

a The majority of *PIK3CA* documented mutations being somatic missense mutations, this table does not include other genetic changes (i.e. gene amplifications, deletions, insertions, etc.).
